# Supplementary material for: Modelling Skylarks (Alauda arvensis) to Predict Impacts of Changes in Land Management and Policy: Development and Testing of an Agent-Based Model
Source: PLoS One. 2013 Jun 6;8(6):e65803. doi: 10.1371/journal.pone.0065803 (PMC3675089; doi:10.1371/journal.pone.0065803)
Supplement: Supporting Information S4 — The skylark ODdox as a zipped archive. (ZIP) [file pone.0065803.s004.zip › Skylark_ODdox/globals_defs.html]

ALMaSS Skylark ODdox: File Members


ALMaSS Skylark ODdox
 2.0


- Main Page
- Related Pages
- Classes
- Files

- File List
- File Members

- All
- Functions
- Variables
- Typedefs
- Enumerations
- Enumerator
- Macros

- \_
- c
- d
- e
- h
- l
- p
- r
- s
- t
- u

### - \_ -

- \_\_3264divide
  : PositionMap.cpp
- \_\_3264minus0
  : PositionMap.cpp
- \_\_3264minus1
  : PositionMap.cpp
- \_\_64bit
  : ALMaSS\_Setup.h
- \_\_CJTDebug\_7
  : skylarks\_all.cpp
- \_\_WEED\_CURVE
  : elements.cpp
- \_CRT\_SECURE\_NO\_DEPRECATE
  : weather.cpp
  , croprotation.cpp
  , plants.cpp
  , pesticide.cpp
  , misc.cpp
  , maperrormsg.cpp
  , rastermap.cpp
  , farm.cpp
  , MovementMap.cpp
  , elements.cpp
  , farm.cpp
  , configurator.cpp
  , hedgebanks.cpp
  , ALMaSS\_CmdLine.cpp
- \_CRTDBG\_MAP\_ALLOC
  : landscape.h
  , PopulationManager.cpp
  , farm.cpp

### - c -

- CFG\_CHANNEL\_BITS
  : pesticide.cpp
- CFG\_CHANNEL\_MAXVAL
  : pesticide.cpp
- CFG\_MAX\_LINE\_LENGTH
  : configurator.h

### - d -

- DEBUG\_NEW
  : configurator.cpp
- DO\_IT\_PROB
  : farmfuncs.cpp

### - e -

- EL\_BUG\_GLUE
  : elements.cpp
- EL\_BUG\_PERCENT\_A
  : elements.cpp
- EL\_BUG\_PERCENT\_B
  : elements.cpp
- EL\_BUG\_PERCENT\_C
  : elements.cpp
- EL\_BUG\_PERCENT\_D
  : elements.cpp
- EL\_BUG\_PERCENT\_Edges\_A
  : elements.cpp
- EL\_BUG\_PERCENT\_Edges\_B
  : elements.cpp
- EL\_BUG\_PERCENT\_Edges\_C
  : elements.cpp
- EL\_BUG\_PERCENT\_G\_A
  : elements.cpp
- EL\_BUG\_PERCENT\_G\_B
  : elements.cpp
- EL\_BUG\_PERCENT\_G\_C
  : elements.cpp
- EL\_BUG\_PERCENT\_SB\_A
  : elements.cpp
- EL\_BUG\_PERCENT\_SB\_B
  : elements.cpp
- EL\_BUG\_PERCENT\_SB\_C
  : elements.cpp
- EL\_BUG\_PERCENT\_WR\_A
  : elements.cpp
- EL\_BUG\_PERCENT\_WR\_B
  : elements.cpp
- EL\_BUG\_PERCENT\_WR\_C
  : elements.cpp
- EL\_BUG\_PERCENT\_WRy\_A
  : elements.cpp
- EL\_BUG\_PERCENT\_WRy\_B
  : elements.cpp
- EL\_BUG\_PERCENT\_WRy\_C
  : elements.cpp
- EL\_BUG\_PERCENT\_WW\_A
  : elements.cpp
- EL\_BUG\_PERCENT\_WW\_B
  : elements.cpp
- EL\_BUG\_PERCENT\_WW\_C
  : elements.cpp
- EL\_BUG\_SLOPE
  : elements.cpp
- EL\_GROWTH\_DATE\_MAGIC
  : elements.cpp
- EL\_GROWTH\_DAYDEG\_MAGIC
  : elements.cpp
- EL\_GROWTH\_PHASE\_SHIFT\_LEVEL
  : elements.cpp
- EL\_HERBICIDE\_DELAYTIME
  : elements.h
- EL\_MAX\_USERSPACE
  : elements.h
- EL\_PLANT\_BIOMASS
  : elements.cpp
- EL\_STRIGLING\_DELAYTIME
  : elements.h
- EL\_TRAMLINE\_DECAYTIME
  : elements.h
- EL\_VEG\_HEIGHTSCALE
  : elements.cpp
- EL\_VEG\_START\_LAIT
  : elements.cpp
- EL\_WEED\_GLUE
  : elements.cpp
- EL\_WEED\_PERCENT
  : elements.cpp
- EL\_WEED\_SLOPE
  : elements.cpp

### - h -

- HB\_MAGIC
  : hedgebanks.cpp
- HB\_MAGIC\_COLOR
  : hedgebanks.cpp
- HB\_MAGIC\_PAINTER\_BIT
  : hedgebanks.cpp

### - l -

- LE\_SIG\_NO\_FUNGICIDE
  : elements.h
- LE\_SIG\_NO\_GROWTH\_REG
  : elements.h
- LE\_SIG\_NO\_HERBICIDE
  : elements.h
- LE\_SIG\_NO\_INSECTICIDE
  : elements.h
- LE\_SIG\_NO\_MOLLUSC
  : elements.h
- LE\_SIG\_NO\_SYNG\_INSECT
  : elements.h

### - p -

- PEST\_DEBUG
  : pesticide.h
- PEST\_GRIDAREA
  : pesticide.h
- PEST\_GRIDSIZE
  : pesticide.h
- PEST\_GRIDSIZE\_POW2
  : pesticide.h
- PEST\_WATER\_CHECK
  : pesticide.h
- PROG\_START
  : farm.h

### - r -

- RV\_CUT\_AUG
  : elements.cpp
- RV\_CUT\_GREEN
  : elements.cpp
- RV\_CUT\_HEIGHT
  : elements.cpp
- RV\_CUT\_JUL
  : elements.cpp
- RV\_CUT\_JUN
  : elements.cpp
- RV\_CUT\_MAY
  : elements.cpp
- RV\_CUT\_OCT
  : elements.cpp
- RV\_CUT\_SEP
  : elements.cpp
- RV\_CUT\_TOTAL
  : elements.cpp
- RV\_MAY\_1ST
  : elements.cpp

### - s -

- SKOPTIMALHABITATSCORE
  : skylarks\_all.cpp
- SV\_INT32
  : pesticide.cpp
- SV\_INT8
  : pesticide.cpp
- SV\_UINT32
  : pesticide.cpp
- SV\_UINT8
  : pesticide.cpp

### - t -

- TLANDSCAPE\_H
  : landscape.h

### - u -

- UNREFERENCED\_PARAMETER
  : farmfuncs.cpp
  , skylarks\_all.h


- Generated on Thu Jan 10 2013 13:15:39 for ALMaSS Skylark ODdox by
   1.8.1.1
